# The benefit of minocycline on negative symptoms of schizophrenia in patients with recent-onset psychosis (BeneMin): a randomised, double-blind, placebo-controlled trial

**DOI:** 10.1016/S2215-0366(18)30345-6

**Published:** 2018-11

**Authors:** Bill Deakin, John Suckling, Thomas R E Barnes, Kelly Byrne, Imran B Chaudhry, Paola Dazzan, Richard J Drake, Annalisa Giordano, Nusrat Husain, Peter B Jones, Eileen Joyce, Emma Knox, Carl Krynicki, Stephen M Lawrie, Shôn Lewis, Danuta M Lisiecka-Ford, Naghmeh Nikkheslat, Carmine M Pariante, Richard Smallman, Andrew Watson, Steven C R Williams, Rachel Upthegrove, Graham Dunn, BeneMin Study team

**Affiliations:** a Neuroscience and Psychiatry Unit, The University of Manchester, Manchester, UK; b MAHSC, The University of Manchester, Manchester, UK; c Division of Neuroscience and Experimental Psychology, The University of Manchester, Manchester, UK; d Division of Psychology and Mental Health, The University of Manchester, Manchester, UK; e Division of Population Health, Health Services Research and Primary Care, The University of Manchester, Manchester, UK; f Greater Manchester Mental Health NHS Foundation Trust, Prestwich, Manchester, UK; g Brain Mapping Unit, Department of Psychiatry, Herchel Smith Building for Brain and Mind Sciences, University of Cambridge, Cambridge, UK; h Neurology Unit, Department of Clinical Neurosciences, University of Cambridge, Cambridge, UK; i Cambridgeshire & Peterborough NHS Foundation Trust, Cambridge, UK; j Centre for Psychiatry, Imperial College London, London, UK; k Tropical Clinical Trials Unit, Liverpool School of Tropical Medicine, Liverpool, UK; l Lancashire Care Early Intervention Service, Accrington, UK; m Department of Psychosis Studies, Institute of Psychiatry, Psychology and Neuroscience, King's College London, London, UK; n Stress, Psychiatry and Immunology Lab & Perinatal Psychiatry, The Maurice Wohl Clinical Neuroscience Institute, King's College London, London, UK; o Department for Neuroimaging, King's College London, London, UK; p Sobell Department of Motor Neurosciences and Movement Disorders, UCL Institute of Neurology, London, UK; q Institute for Applied Clinical Sciences, Keele University, Guy Hilton Research Centre, Stoke-on-Trent, UK; r Institute for Mental Health, University of Birmingham, Birmingham, UK; s Division of Psychiatry, Centre for Clinical Brain Sciences, University of Edinburgh, Edinburgh, UK

## Abstract

**Background:**

The antibiotic minocycline has neuroprotective and anti-inflammatory properties that could prevent or reverse progressive neuropathic changes implicated in recent-onset schizophrenia. In the BeneMin study, we aimed to replicate the benefit of minocycline on negative symptoms reported in previous pilot studies, and to understand the mechanisms involved.

**Methods:**

In this randomised, double-blind, placebo-controlled trial, we recruited people with a schizophrenia-spectrum disorder that had begun within the past 5 years with continuing positive symptoms from 12 National Health Service (NHS) trusts. Participants were randomly assigned according to an automated permuted blocks algorithm, stratified by pharmacy, to receive minocycline (200 mg per day for 2 weeks, then 300 mg per day for the remainder of the 12-month study period) or matching placebo, which were added to their continuing treatment. The primary clinical outcome was the negative symptom subscale score of the Positive and Negative Syndrome Scales (PANSS) across follow-ups at months 2, 6, 9, and 12. The primary biomarker outcomes were medial prefrontal grey-matter volume, dorsolateral prefrontal cortex activation during a working memory task, and plasma concentration of interleukin 6. This study is registered as an International Standard Randomised Controlled Trial, number ISRCTN49141214, and the EU Clinical Trials register (EudraCT) number is 2010-022463-35I.

**Findings:**

Between April 16, 2013, and April 30, 2015, we recruited 207 people and randomly assigned them to receive minocycline (n=104) or placebo (n=103). Compared with placebo, the addition of minocycline had no effect on ratings of negative symptoms (treatment effect difference −0·19, 95% CI −1·23 to 0·85; p=0·73). The primary biomarker outcomes did not change over time and were not affected by minocycline. The groups did not differ in the rate of serious adverse events (n=11 in placebo group and n=18 in the minocycline group), which were mostly due to admissions for worsening psychiatric state (n=10 in the placebo group and n=15 in the minocycline group). The most common adverse events were gastrointestinal (n=12 in the placebo group, n=19 in the minocycline group), psychiatric (n=16 in placebo group, n=8 in minocycline group), nervous system (n=8 in the placebo group, n=12 in the minocycline group), and dermatological (n=10 in the placebo group, n=8 in the minocycline group).

**Interpretation:**

Minocycline does not benefit negative or other symptoms of schizophrenia over and above adherence to routine clinical care in first-episode psychosis. There was no evidence of a persistent progressive neuropathic or inflammatory process underpinning negative symptoms. Further trials of minocycline in early psychosis are not warranted until there is clear evidence of an inflammatory process, such as microgliosis, against which minocycline has known efficacy.

**Funding:**

National Institute for Health Research Efficacy and Mechanism Evaluation (EME) programme, an MRC and NIHR partnership.

## Introduction

Antipsychotic drugs in schizophrenia can effectively promote remission of so-called positive psychotic symptoms, such as delusions, hallucinations, and disorganised speech. Nevertheless, a poor quality of life with impaired social and occupational functioning is common despite continuing medical, rehabilitative, and psychological treatment. A major underlying problem is the persistence of so-called negative symptoms: social withdrawal, self-neglect, and loss of emotional responsiveness and motivation, together with mild cognitive impairment.^1^ The pathogenesis of the negative syndrome is unknown and the scarcity of validated CNS targets probably accounts for the failure of many attempts to find effective medical treatments. Early studies plausibly attributed negative symptoms to the static neurodevelopmental cerebral atrophy associated with the disorder.^2^ However, later MRI studies reported evidence of a progressive loss of grey matter occurring before onset of psychosis and continuing in early years of psychosis.^3^, ^4^, ^5^ That early treatment with a neuroprotective drug might prevent such a process and its symptomatic consequences was the prime motivation for this study and its precursor.^6^

**Research in context**

**Evidence before this study**

The antibiotic minocycline has neuroprotective and anti-inflammatory actions that have attracted attention as potential treatments for several neurodegenerative disorders, including a possible neuropathic process in schizophrenia. Several case reports, open-label studies, and small controlled trials have claimed benefit, particularly for negative symptoms such as apathy and social withdrawal. The negative syndrome is little improved by current treatments and predicts poor social and occupational functioning. Emerging evidence of an inflammatory process in schizophrenia and depression has reinforced interest in minocycline and other anti-inflammatory drugs as a new direction in psychiatric treatment. We searched PubMed with the terms “minocycline” and “schizophrenia” or “psychosis”, filtering for “clinical trial”, on Aug 20, 2018. We found six studies in English, all involving minocycline as an adjunctive treatment compared with placebo, five of which targeted negative symptoms. Two early studies provided proof of concept for the BeneMin trial, funded in parallel by the same funder. A 12-month trial in Pakistan and Brazil had a larger sample size of more than 45 patients who completed the study per group. Minocycline showed efficacy on negative symptoms in both centres but the benefit was significantly less in the Pakistan subsample. Efficacy in a study from Tel Aviv, Israel, was assessed at 22 weeks and based on 23 patients who completed the study receiving minocycline and eight receiving placebo, which were initiated during acute psychosis. Two studies have subsequently reported substantially greater improvement with minocycline than placebo over 8 weeks (in Iran) and 16 weeks (in China), in stable patients taking risperidone. The Iranian study was one of 24 mostly positive trials of various adjunctive drugs on negative symptoms published by the same study group in less than 10 years. A study from a second Iranian group found no significant benefit over 8 weeks in patients on risperidone. A US study in patients who were clozapine resistant found no benefit of minocycline over placebo on positive symptoms (the primary outcome) or negative symptoms.

**Added value of this study**

To our knowledge, the BeneMin study is the largest double-blind, placebo-controlled trial of minocycline to date. It addresses the therapeutic potential of minocycline in recent-onset schizophrenia and also probed its potential neuroprotective and anti-inflammatory mechanism of action. Strengths of the study include the large sample size, effective blinding, adequate retention, and the 12-month exposure to minocycline. The results are decisively negative, with no evidence of deterioration in negative symptoms or indices of inflammation or grey-matter loss, and no evidence that minocycline prevents or ameliorates negative symptoms or inflammatory or neuropathic processes. Our results are generalisable to patients with schizophrenia treated with antipsychotic medication within 5 years of illness onset.

**Implications of all the available evidence**

Minocycline should not be used in the early adjunctive treatment of schizophrenia. Further trials are not indicated in this population, unless a potentially responsive inflammatory or progressive subtype can be identified. Convergent PET imaging studies show little evidence that microglial inflammation occurs in drug-free patients with schizophrenia; together with the absence of effect of minocycline, the results suggest that active neuroinflammation involving microglial activation and neuropathology is not a pervasive feature of the first years of schizophrenia.

We initially selected minocycline because of preclinical evidence of neuroprotective effects in experimental models of stroke and Huntington's disease, related to its ability to inhibit caspases and the apoptotic pathway.^7^ Preliminary clinical trials had also reported possible benefit of minocycline in patients with stroke and Parkinson's disease,^8^, ^9^ but with no benefit on neurological symptoms in Huntington's disease^10^ and worsening in patients with motor neurone disease.^11^ Early open-label clinical studies had reported benefit in patients with schizophrenia.^12^ Interest in the possible therapeutic benefit of the anti-inflammatory actions of minocycline grew with increasing evidence for raised circulating cytokine concentrations in schizophrenia.^13^ Furthermore, increased PET radioligand binding to the translocator protein, expressed in activated microglia, had been reported in patients with recent-onset schizophrenia.^14^ Whether microglia are activated in an inflammatory state in schizophrenia has become a key research question. Minocycline is widely used as a pharmacological inhibitor of microglial activation in experimental animal studies,^15^ and its possible efficacy in schizophrenia is a key test of microglial involvement in the disorder.

The precursor to this present study was a two-centre trial in Brazil and Pakistan.^6^ 94 people with schizophrenia taking stable medication completed 12 months of add-on treatment with placebo or minocycline.^6^ Negative symptoms improved significantly more in the minocycline group than in the placebo group. In a study in Tel Aviv,^16^ relapsed patients were randomly assigned to placebo (n=18) or minocycline (n=36) within 2 weeks of treatment initiation. Negative symptoms increased after 3 months in the placebo group but not in the minocycline group, and there was no difference at 6 months. In the BeneMin study, we aimed to replicate the therapeutic effects of minocycline in a large sample, to characterise the time course over 12 months, and to test the hypothesis that any benefit of minocycline on negative symptoms is due to neuro-protection, possibly involving anti-inflammatory actions. The 12-month design aimed to allow sufficient time for the emergence of negative symptoms and for a detectable loss of grey-matter volume, on the basis of findings from the 1-year structural imaging study of Lieberman and colleagues.^4^

## Methods

### Study design and participants

BeneMin was a multicentre, double-blind, randomised, placebo-controlled study involving participants recruited from 12 UK National Health Service trusts. The hypotheses and full study protocol have been published.^17^ The North West Manchester Research Ethics Committee (reference number 11/NW/0218) approved the study. Full details of the study will be published elsewhere.^18^

Eligible patients were in a first episode of schizophrenia, schizophreniform, or schizoaffective psychosis (DSM-IV) as assessed by the research team, with continuing positive symptoms defined by a score greater than 2 for one or more items (P1 delusions, P2 conceptual disorganisation, P3 hallucinatory behaviour, or P6 suspiciousness) of the Positive and Negative Syndrome Scale (PANSS), were within 5 years of onset of symptoms judged sufficient to meet diagnostic criteria, had an intelligence quotient (IQ) greater than 70 as assessed by the Wechsler Test of Adult Reading (WTAR), and were able to understand and give written informed consent. Participants were required to be taking stable antipsychotic treatment from a mental health-care team.

Participants were excluded if they had a current diagnosis of substance misuse, had a current serious risk of suicide or violence, had used tetracycline antibiotics within 2 months of baseline visit or had a history of sensitivity or intolerance to an antibiotic, had a relevant current or past medical disorder or were pregnant or breastfeeding, or met MRI exclusion criteria.

Recruitment followed the previously validated procedures of PsyGrid.^19^ The clinical team made the first approach. The participant's responsible medical officer or care coordinator completed a diagnostic and eligibility criteria checklist and elicited permission for contact from the research team. Written informed consent was recorded.

### Randomisation and masking

Patients were randomly assigned with an automated permuted blocks algorithm, and were stratified by pharmacy. openCDMS (a now discontinued open-source clinical data management system) allocated the patient to a treatment group at randomisation, emailed the local pharmacy to identify the numbered treatment kit of 3 months' supply to be dispensed, and recorded when a kit was dispensed. Emergency unblinding using openCDMS was governed by a standard operating procedure. The data management and ethics committee statistician could, but did not, request access to unblinded data on openCDMS operated by the informatics department at the University of Manchester. Coded supplies of placebo and active minocycline were manufactured and distributed to local pharmacies by Catalent and tracked by openCDMS.

### Procedures

Patients took two 100 mg capsules of modified-release minocycline or matching placebo every day for 2 weeks, then three 100 mg tablets per day for the remainder of the 12-month study period, in addition to standard therapy and routine care.

For the schedule of assessments, see the appendix. Research assistants obtained informed consent to the procedures described in a patient information leaflet about the trial. At screening, the research assistant applied a diagnostic checklist to the case notes and, on the basis of a Mini-International Neuropsychiatric Interview, reached a consensus diagnosis with the responsible medical officer and confirmed the presence of psychotic symptoms. Blood was taken for renal and liver function tests (if not available from routine care) and urine for drugs and pregnancy tests. At the randomisation visit, all baseline efficacy, MRI scans, and mechanistic outcomes were recorded, together with assessments of side-effects and medication adherence (appendix).

Efficacy, side-effects, and adherence assessments were repeated at follow-up visits at 2, 6, 9, and 12 months. MRI and cognitive function tests were repeated at 12 months. Blood samples to test for high-sensitivity C-reactive protein (hs-CRP) and cytokines were repeated at months 6, 9, and 12. Trial medication ceased at 12 months. Clinical ratings were repeated at 15 months to check for rebound symptoms after withdrawal from trial medication. Project research assistants had regular training and harmonisation discussions at teleconferences every 2 weeks and at away days every 6 months. We used reference PANSS interview videos to maintain and monitor inter-rater reliability.

Bloods for plasma inflammatory markers were aliquoted and frozen within 4 h. hs-CRP was measured with the Cormay anti-CRP antibody (PZ Cormay SA, Lomianki, Poland) sensitised to latex particles. Interleukin 6 (IL-6) was assayed with Meso Scale Discovery V-PLEX sandwich immunoassays (Rockville, MD, USA). Assays were done at King's College London, supervised by PD.

JS coordinated the MRI sequences, which were based on the previous NeuroPsygGrid multi-centre validation and reliability study^20^ comprising three-dimensional T1-weighted magnetisation-prepared rapid gradient-echo (MPRAGE/SPGR), proton density and T2-weighted dual echo (PD/T2), and T2*-weighted gradient echo planar imaging during N-back working memory task and resting state. Scans were transferred to and analysed by JS. Cognitive function testing across centres was coordinated and monitored by EJ.

We assessed adherence to trial medication using a seven-point semi-structured interview rating about attitude to and frequency of taking medication (appendix). As a minor amendment to the protocol, we also assayed the 6-month and 12-month plasma samples for minocycline after the study, using high-performance liquid chromatography.^18^

The trial was overseen by an independent Trial Steering Committee that included a service user. The committee received reports from the data monitoring committee to determine whether there was evidence of harm to participants from active medication, harm from withholding an overwhelmingly beneficial treatment from those on placebo, or feasibility or ethical barriers to continuing the trial in its current or modified form to achieve its stated objectives.

### Outcomes

The primary clinical outcome was overall severity of negative symptoms of psychosis or their change over time, as measured by the negative symptom subscale score in PANSS, at months 2, 6, 9, and 12.

The secondary clinical outcomes were positive symptom subscale and total score of PANSS; Calgary Depression Scale for Schizophrenia (CDSS); Global Assessment of Functioning; Social Functioning Scale, a self-rating scale assessing social functioning in domains such as social engagement, interpersonal behaviour, pro-social activities, independence or employment; cognitive function as measured by short Wechsler Adult Intelligence Scale III for patients with schizophrenia (current IQ), which included the subtests Information, Arithmetic, Block Design and the Digit Symbol test of processing speed; WTAR for pre-morbid IQ; Verbal Fluency, and Auditory-Verbal Learning Task Test trials 1–5; bodyweight and body-mass index (BMI); and current and previous drug treatment.

The primary biomarker outcomes were medial prefrontal grey-matter volume (measured with structural MRI), circulating IL-6 concentration, and N-back performance and blood-oxygen-level-dependent imaging (BOLD) response in dorsolateral-prefrontal cortex (measured with functional MRI).

The secondary biomarker outcomes were regional grey-matter volume, hs-CRP and major cytokines (which will be reported elsewhere), and resting state connectivity.

The general medical care of participants was the responsibility of the mental health-care team and general practitioner. Haematology, renal, and liver blood screens were arranged if none was available within the past 3 months. Any new physical or subjective symptoms were recorded as adverse events or reactions, or serious adverse events with standard reporting procedures. Assessments of neurological and non-neurological side-effects of antipsychotic drugs are detailed in the appendix.

### Statistical analysis

The study was designed to produce clinical and biomarker data for 170 patients completing 1 year of placebo or minocycline add-on treatment (n=85 per group). This sample would have 90% power to detect a standardised effect size of 0·5 in the primary clinical outcome (eg, a group difference in negative symptom scores of 3 units, assuming the within-group SD is equal to 6) using a two-tailed *t* test (at p<0·05). On the basis of the NeuroPsyGrid five-site imaging data,^20^ the minimal detectable difference in grey matter is 2% at 80% power at this sample size.

The statistical analysis was overseen by study statistician (GD) to a plan agreed by the Trial Steering Committee. There were no interim analyses and all analyses were carried out after the collection of the final outcome measures. We did all statistical analyses of the clinical, cognitive, and biomarker outcomes using Stata (version 14). When the primary analyses were complete, the treatment code was revealed.

We used baseline variables to check balance of the randomised groups. We reported treatment effects using 95% CI, supplemented by their associated p values. Treatment effects are the difference between group means (minocycline minus placebo) attributable to treatment after controlling for baseline and academic centre. The treatment effect is reported as an average outcome across follow-up timepoints for the minocycline group minus the average outcome for the placebo group. Thus, for severity scores, negative treatment effects indicate a beneficial effect of minocycline (less severity than placebo). For function scores in which a high score is a good outcome, a beneficial effect of minocycline would be indicated by a positive treatment effect.

We estimated treatment effects by using a random effects regression model (using Stata's xtreg command) after allowing for time of follow-up (2, 6, 9, or 12 months, treated as a categorical variable), academic centre, and baseline severity of negative symptoms. We assessed the effect of time of follow-up on treatment efficacy by the interactions of treatment by time and treatment effects at the four follow-up times, estimated separately. If there was no significant interaction between the treatment effect and time, we removed the interaction from the model and estimated treatment efficacy common to all four follow-up times. All models contained interactions of centre by time and baseline severity by time. An exploratory analysis examined treatment effects in each academic centre separately. We assessed the sensitivity of efficacy estimates to the effects of poor adherence with medication and other covariates on loss to follow-up using descriptive summary statistics and the use of inverse probability weighting.^18^

We visually inspected all MRI scans for quality and possible acquisition artefacts. Subsequently, we used standardised method-specific temporal and spatial processing with the FSL software library on each scan (appendix). We analysed MPRAGE/SPGR and PD/T2 images using sequence-specific voxel-based morphometry. We extracted mean grey-matter volumes from bilateral medial prefrontal cortex defined independently by an atlas.^21^ We extracted mean BOLD responses corresponding to orthogonal contrasts of working memory (1-back and 2-back *vs* 0-back; 2-back *vs* 1-back) from atlas regions corresponding to the dorsolateral prefrontal cortex. We used a random effects model to estimate the main effects of treatment group and time and their interaction on the grey-matter volume and BOLD responses, as with other outcome variables. Participant sex and age, and MRI acquisition centre were included as covariates. The threshold of significance was set at a p value of 0·05 or less. This study is registered as an International Standard Randomised Controlled Trial, number ISRCTN49141214, and the EU Clinical Trials register (EudraCT) number is 2010-022463-35I.

### Role of the funding source

The funder of the study had no role in study design, data collection, data analysis, data interpretation, or writing of the report. The corresponding author had full access to all the data in the study and had final responsibility for the decision to submit for publication.

## Results

**Figure 1:**
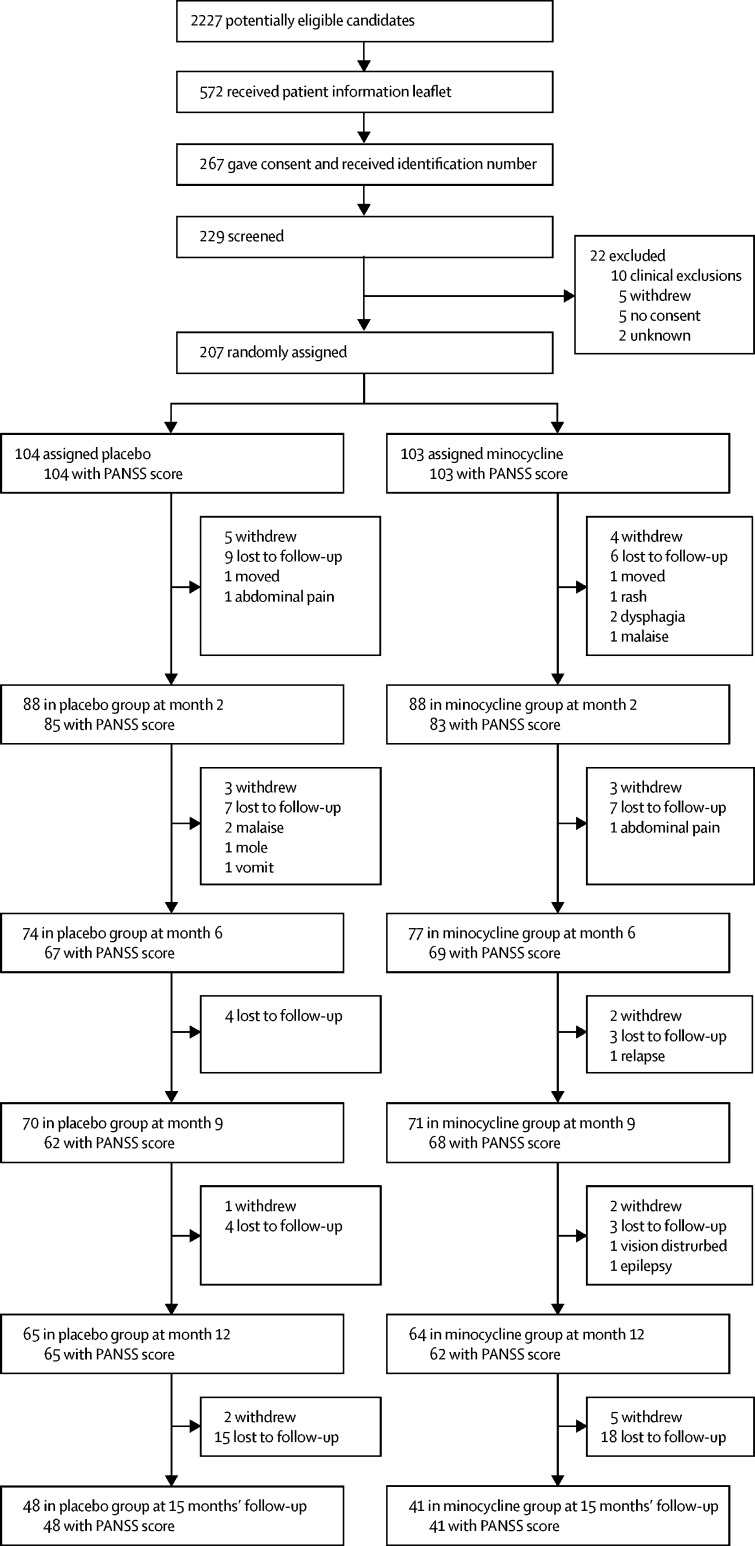
Trial profile

Between April 16, 2013, and April 30, 2015, 207 participants were recruited. The last 12-month visit occurred on June 9, 2016. Figure 1 shows the numbers of participants recruited and remaining in the study, including those who missed an assessment but attended a subsequent one, up to and including the 12-month visit (the end of the treatment phase). The number of participants seen at each appointment for whom PANSS scores were recorded is also shown (figure 1). Overall, 78 (38%) of 207 randomly assigned participants dropped out during the 12-month treatment phase. In 63 (81%) of these participants, the reasons were either a participant's request or loss to follow-up. Scores for the primary outcome measure at 6 months were available for 136 (66%) participants and, at 12 months, 127 (61%) participants. The numbers of participants dropping out were evenly split throughout: at 15 months, 11 participants in the placebo group versus 16 in the minocycline grup had withdrawn, and 39 versus 37 were lost to follow-up. There were 12 allocation centres, each of which had a collaborating pharmacy linked to OpenCDMS and one of the six universities of the principal investigators and research assistants. Each centre allocated a similar number of participants to placebo and minocycline. About a quarter of each group were prescribed long-acting depots. Oral medication was almost entirely confined to second-generation antipsychotics (n=86 in the placebo group, n=90 in the minocycline group), most frequently olanzapine, followed in equal measure by risperidone, aripiprazole, and amisulpride.^18^

The two treatment groups did not differ in terms of age, sex, or any baseline measures of the primary and other major outcome variables (table 1). Mean total PANSS scores of 67 (minocycline group) and 69 (placebo group) indicated a mild-to-moderate level of symptom severity.^22^ Global Assessment of Functioning scores in the mid-50s at baseline indicate a moderate level of severity of impairment of social and occupational function (table 1). This finding was corroborated by low subscale scores for the Social Functioning Scale, particularly in spontaneous recreational activity and pro-social activity (appendix). The mean CDSS was greater than 5 in both groups, with 37 (36%) participants in the placebo and 39 (38%) in the minocycline group having scores greater than 6, indicating a possible major depressive episode (data not shown).^23^ In both groups, premorbid IQ, as assessed by the WTAR, was just less than 100, whereas current IQ was about 5 points less (table 1). 13 participants had increased hs-CRP concentrations (>10 mg/L) at baseline, indicating a probable recent viral or possibly bacterial infection, otherwise baseline IL-6 and hs-CRP concentrations were unremarkable (table 1).

**Table 1:** Baseline demographics and outcome variables

		**Placebo**		**Minocycline**	
		n	Mean (SD)	n	Mean (SD)
Sex					
	Male	73	..	77	..
	Female	30	..	27	..
Age, years		101	25·7 (5·1)	103	25·5 (5·2)
PANSS score					
	Negative symptom subscale score	104	16·8 (5·5)	103	17·7 5·9)
	Positive symptom subscale score	104	17·3 (5·3)	103	16·3 (4·1)
	Total PANSS score	103	69·3 (15·4)	103	67·1 (13·2)
CDSS score		103	5·5 (5·0)	103	5·2 (4·3)
GAF score		103	56·2 (11·6)	102	55·5 (9·1)
Weight, kg		101	86·8 (25·3)	97	82·6 (19·6)
Body-mass index		101	28·7 (7·6)	96	27·1 (6·2)
Processing speed		91	52·8 (16·8)	95	58·0 (16·7)
Current IQ		100	89·2 (15·9)	101	91·2 (14·0)
Premorbid IQ		98	95·4 (19·8)	100	97·7 (1·7)
Medial prefrontal cortex grey-matter volume (cc)					
	Left grey-matter volume	88	5·7 (0·8)	94	5·6 (0·7)
	Right grey-matter volume	88	4·6 (0·7)	94	4·6 (5·8)
N-back BOLD activation (% change)					
	1-back plus 2-back *vs* 0-back	88	0·12% (1·25)	94	−0·02% (1·48)
	2-back *vs* 1-back	88	0·10% (1·23)	94	−0·04% (1·54)
Cytokine IL-6, pg/mL		100	0·84 (0·64)	101	0·69 (0·46)
hs-CRP, mg/L		100	3·83 (5·45)	101	3·08 (3·82)

PANSS=Positive and Negative Syndrome Scales. CDSS=Calgary Depression Scale for Schizophrenia. GAF=Global Assessment of Functioning. IQ=intelligence quotient. BOLD=blood-oxygen-level-dependent imaging. IL-6=interleukin 6. hs-CRP=high-sensitivity C-reactive protein.

**Figure 2:**
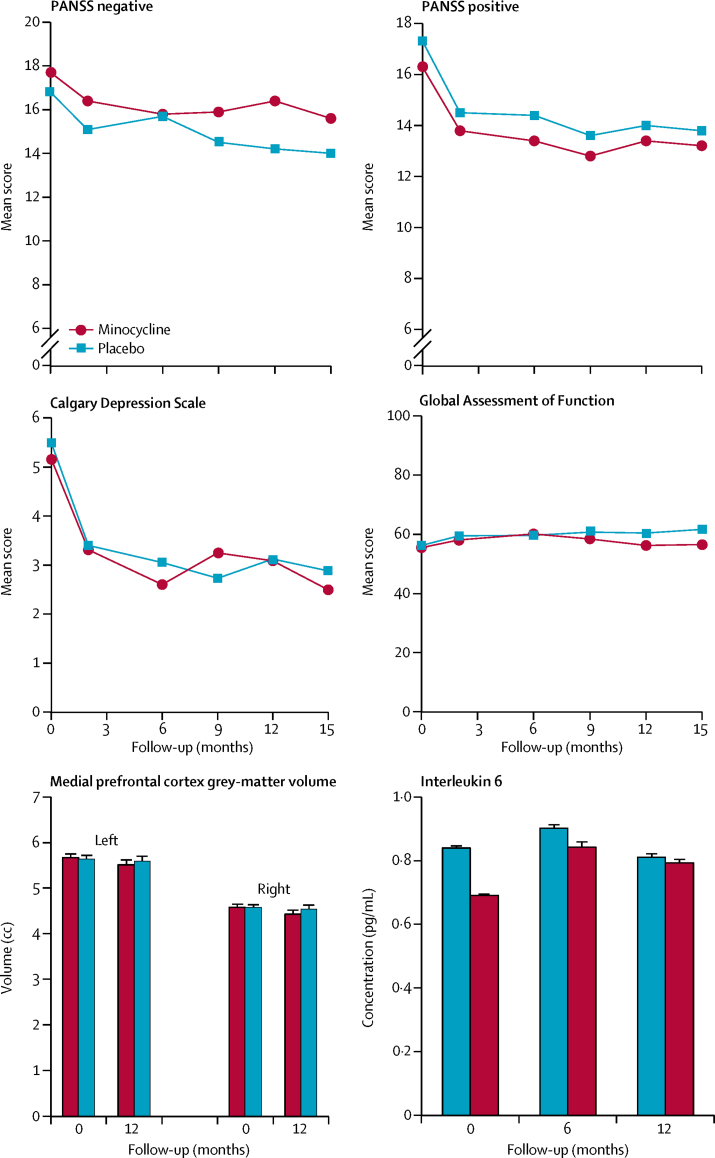
Main outcome measures for minocycline and placebo groups The minimum scores on Positive and Negative Syndrome Scale (PANSS) subscales is 7.

Minocycline had no discernible influence on any clinical outcome variable in terms of direction, magnitude, or statistical significance. The observed case means were closely similar in both groups (figure 2). There were no significant treatment effects on measures assessed at all follow-up timepoints (table 2). Negative symptom subscale scores were similar in each group throughout the study, with no tendency to worsen in the placebo group as we had expected. In an exploratory analysis by the trial statistician, treatment effects did not differ in participants with above and below median hs-CRP or IL-6 concentrations at baseline. There was no indication of a rebound worsening of negative symptoms recorded at 15 months, after cessation of treatment at 12 months, in either group. Mean scores for negative symptoms, positive symptoms, and depression symptoms improved in both groups across the trial (appendix). There was moderately good agreement (intra-class correlation 0·7) between the seven principal research assistants of their negative PANSS scores in up to 11 reference videos of SCI-PANSS interviews.

**Table 2:** Summary of best estimates of treatment effects across all follow-up timepoints

	**Estimate (95% CI); p value**	**Standard error**
**Primary outcome**		
Negative symptoms (PANSS)*	−0·19 (−1·23 to 0·85); p=0·73	0·53
**Clinical outcomes**		
Positive symptoms (PANSS)*	−0·19 (−1·12 to 0·73); p=0·68	0·47
Total symptoms (PANSS)*	−0·58 (−3·75 to 2·59); p=0·72	1·62
CDSS score	−0·06 (−0·84 to 0·72); p=0·88	0·40
GAF score*	2·71 (−1·57 to 6·98); p=0·21	2·15
SFS Withdrawal†	−0·24 (−1·03 to 0·55); p=0·55	0·40
SFS Relations†	−0·02 (−0·55 to 0·51); p=0·94	0·27
SFS Independence-Performance†	−0·78 (−2·53 to 0·97); p=0·38	0·89
SFS Recreation†	−0·91 (−2·65 to 0·82); p=0·30	0·89
SFS Prosocial Activities†	0·19 (−2·25 to 2·62); p=0·88	1·24
SFS Independence-Competence†	−0·49 (−1·79 to 0·81); p=0·46	0·67
SFS Employment†	−0·12 (−0·95 to 0·71); p=0·78	0·43
Processing speed‡	−2·14 (−6·63 to 2·35); p=0·35	2·26
Current IQ‡	−0·56 (−3·59 to 2·47); p=0·72	1·53
Weight‡	2·71 (−1·57 to 6·98); p=0·21	2·15
**Biomarker outcomes**		
Left grey-matter volume‡	−0·09 (−0·30 to 0·12); p=0·40	0·11
Right grey-matter volume‡	−0·07 (−0·21 to 0·08); p=0·34	0·07
N-back BOLD activation (% change): 1-back plus 2-back *vs* 0-back	−0·66 (−1·53 to 0·20); p=0·13	0·43
N-back BOLD activation (% change): 2-back *vs* 1-back	−0·57 (−1·40 to 0·26); p=0·18	0·41
Interleukin 6†	0·07 (−0·12 to 0·26); p=0·46	0·10
High-sensitivity C-reactive protein†	1·72 (−1·42 to 4·85); p=0·28	1·60

PANSS=Positive and Negative Syndrome Scales. CDSS=Calgary Depression Scale for Schizophrenia. GAF=Global Assessment of Functioning. SFS=Social Functioning Scale. IQ=intelligence quotient. BOLD=blood-oxygen-level-dependent imaging.

* Follow-up at months 2, 6, 9, and 12.

† Follow-up at months 6 and 12.

‡ 12-month follow-up.

There were no significant effects of treatment on primary or secondary biomarker outcomes. Medial prefrontal grey-matter volume did not show the expected decrease over 12-months in either group (figure 2). N-back performance (% correct) improved by 10% in both groups over 12 months (appendix). The task engaged the predicted fronto-parietal executive network with greater BOLD activation in the more difficult 2-back versus 1-back condition (appendix). There were no effects of treatment on BOLD responses or performance. Circulating hs-CRP and IL-6 concentrations were stable and unaffected by treatment (figure 2; appendix). hs-CRP and IL-6 intercorrelated, and both correlated with BMI (p<0·005) at baseline and at 6 and 12 months. There were no systematic hs-CRP or IL-6 correlations with clinical PANSS subscale scores or Calgary depression self-ratings (data not shown).

Ratings on the seven-point adherence scale decreased throughout the study; from approximately 90% fully or partially engaged and compliant at 2 months, to 80% at 6 months, 75% at 9 months, and 70% at 12 months (appendix). The planned sensitivity analysis showed that failure to find a beneficial effect of minocycline is unlikely to have arisen from poor compliance and subsequent loss to follow-up.^18^ Minocycline was assayed in 56 plasma samples from the minocycline group at 6 months and was detectable in 32 (57%). Analysis of the effect of treatment on PANSS subscales in the 32 participants with detectable minocycline concentrations did not reveal any benefit of minocycline versus placebo at 6 months.

The prevalence of extrapyramidal side-effects was low and there were no group differences (appendix). There were 67 adverse events in the placebo group and 60 events in the minocycline group (appendix). The most common were gastrointestinal (n=12 in the placebo group, n=19 in the minocycline group), psychiatric (n=16 in placebo group, n=8 in minocycline group), nervous system (n=8 in the placebo group, n=12 in the minocycline group), and dermatological (n=10 in the placebo group, n=8 in the minocycline group).

Admission to hospital was classified as a serious adverse event. All admissions were for intensification of psychosis, in nearly all cases associated with stopping antipsychotic medication. Some were repeat admissions such that 15 admissions occurred in the minocycline group (n=10 patients) and ten admissions occurred in the placebo group (n=6 patients). One patient in the placebo group was admitted with deep-venous thrombosis. No deaths occurred during the study.

## Discussion

There is no evidence from the BeneMin study that minocycline treatment for up to 1 year has an effect on the progression or severity of negative symptoms of schizophrenia within the first 5 years of treatment onset. This study followed the design used in a previous two-centre, placebo-controlled, randomised controlled trial in Pakistan and Brazil, done by Chaudhry and colleagues,^6^ in which minocycline improved negative symptoms when added to treatment as usual for 1 year. Participants in the trial by Chaudhry and colleagues had greater PANSS negative symptom subscale scores (n=22) at baseline than in the present study (n=17). However, there was no evidence of an interaction between treatment and baseline in the BeneMin study. The difference between studies might simply reflect chance variation. The benefit of minocycline in the Tel Aviv study^16^ was also small and offset by the fact that it was not detected in the PANSS ratings but only in the Scale for the Assessment of Negative Symptoms, and there were small numbers (16 patients *vs* 9 controls) remaining in the study at primary outcome assessment. A third study^24^ that reported a large effect in patients with recent-onset schizophrenia was an outlier in a meta-analysis by Xiang and colleagues.^25^ Their meta-analysis included five trials in patients with more chronic illness and overall there was a significant benefit of minocycline on negative symptoms. However, the variability in outcome and divergence between countries make for appreciable uncertainty. To our knowledge, BeneMin is the largest study to date and is unequivocally negative. We discuss three possible explanations for the negative result: absence of CNS inflammation after the acute episode, ineffectiveness of minocycline in CNS inflammation, and limitations of the study.

The lack of effect of minocycline in the present study could indicate that the canonical feature of neuropathic inflammation—activation of microglia—was not present or had ceased by the time participants were recruited, within 5 years of presentation with schizophrenia. We found no evidence of progressive loss of grey matter over 1 year, there was no deterioration in cognitive function, and there was no evidence of systemic inflammation from hs-CRP and IL-6 concentrations. In keeping with these results, a meta-analysis of recent PET imaging studies^26^ found no evidence of increases in translocator protein (TSPO) radioligand binding to activated microglia in early or established schizophrenia. This finding contrasts with two reports of increased microglial activation in depression^27^, ^28^ and in neurodegenerative diseases.^29^ Indeed, reduced TSPO binding was a consistent finding in drug-free or minimally treated patients with schizophrenia.^26^ This important and replicated psychosis-specific abnormality of microglial function is, at face value, a logical explanation for the lack of effect of minocycline. It could indicate impaired microglial surveillance and clearance of dysfunctional, redundant, or pathological synapses in schizophrenia, rather than neuroinflammation. However, the precise implications of low TSPO binding for microglial function and for the neuroinflammatory hypothesis of schizophrenia are not yet clear, and this is an active research area.

Increased circulating hs-CRP and IL-6 concentrations compared with controls have been consistently reported in schizophrenia, depression, and other psychiatric disorders. BeneMin has no control group, but no mean concentrations were less than reported norms^30^ and were lower than in previous investigations in schizophrenia.^31^ Samples were processed and frozen within 4 h of venepuncture. The collection was sufficiently consistent to show the well known influence of BMI on circulating concentrations. There was no effect of minocycline on peripheral circulating hs-CRP or IL-6 concentrations, and these concentrations did not correlate with the severity of negative or other symptoms. Remarkably, there appears to be only one report of minocycline effects on circulating cytokines in PubMed searches. In a study in patients with rheumatoid arthritis,^32^ IL-6 concentrations decreased during 6 months in the minocycline treatment group but not in the placebo group. Whether the between-group difference was significant was not reported. No such changes were observed in the BeneMin study. In future studies targeting inflammation, it will be important to preselect patients who have increased circulating hs-CRP or cytokine concentrations, or other evidence of inflammation.

The failure to observe loss of grey matter casts doubt on the duration or generality of losses reported in previous studies. Evidence suggests that loss of grey-matter volume in the prodromal phase peaks within the first years of psychosis.^4^, ^33^ It seems increasingly likely that some of the loss of grey-matter volume is due to initiation of antipsychotic drug treatment in recent-onset psychosis, and this is more marked with first-generation antipsychotic drugs than with second-generation drugs.^33^ Indeed, there is evidence that second-generation antipsychotic drugs have neuroprotective and anti-inflammatory actions. Less than 5% of the BeneMin patients were taking first-generation antipsychotic drugs It seems plausible, therefore, that any late developmental or antipsychotic-related changes in grey-matter volume had plateaued by the time of recruitment. The improving course of symptoms and stability of biomarkers in the study could reflect the benefit of continuing contact with early intervention services and adherence to treatment.

It is possible that minocycline is an ineffective antimicroglial drug in humans, hence its lack of effect in BeneMin. Indeed, no new evidence has emerged of the benefit of minocycline in preventing or slowing neurodegenerative disease in randomised controlled trials since those noted at the beginning of BeneMin research, except for the improvements reported over placebo in 142 patients with multiple sclerosis.^34^ However, the precise role of microglia in the initiation and continuation of these neurodegenerative disorders is not entirely clear. Furthermore, two randomised controlled trials report beneficial effects of minocycline in depression,^35^, ^36^ in which there is evidence for microglial activation from PET TSPO binding.^27^, ^28^

The aim of the study was to determine whether minocycline protects against the development of negative symptoms during 1 year. The design involved a compromise between early neuroprotective treatment in a first episode, with continuing positive symptoms when a putative neuropathic process might be most active, and allowing sufficient time for the process to produce negative symptoms. We chose 1 year on the basis of reports that observable changes in brain structure occur over 1 year in first-episode psychosis.^4^ However, evidence from longitudinal studies in first-episode psychosis^37^ challenge the premise that a 1-year follow up would be sufficient; they indicate that negative symptoms lessen as positive symptoms stabilise, and remain low and stable for up to 2 years in patients remaining in services and treatment. Indeed, the greatest predictor of follow-up in BeneMin was the baseline score, suggesting that early influences on negative symptoms had already operated before the minocycline trial.

Fewer participants were retained in the study for 12 months (64 in minocycline group, 65 in placebo group) than the planned group size of 85. This outcome mainly reflected participant choice and was not clearly different between the two groups. However, retention was more than 80% at month 2, the standard duration for trials of antipsychotic efficacy, and more than 70% at 6 months, by which time major benefits against an inflammatory disorder might be observable.

Poor treatment adherence in one group or another is a further potential source of bias, but there was no between-group difference in self-rated attitude and adherence to trial medication that could account for a failure to observe a benefit of minocycline.^18^ Furthermore, exploratory analyses found no evidence of trends to efficacy compared with placebo in those with maximum adherence scores or in those with detectable minocycline in plasma at 6 or 12-months.

Poor training or reliability between raters is a potential mechanism for a false negative study. In BeneMin, extensive training and discussions of ratings continued throughout the trial. A single research assistant recruited and tested most of the participants in each centre because staff turnover was low. This approach would increase consistency and sensitivity to change within each centre. Agreement between the seven principal research assistants of negative scores in up to 11 reference videos of SCI-PANSS interviews produced an intra-class correlation of 0·7. Centre was included as a factor in estimating all treatment effects, but none were statistically significant. Furthermore, there were no effects of treatment in any centre when considered separately. Since treatment effects do not vary between centres, it seems unlikely that beneficial effects of minocycline were obscured by marked deviation in rating or other unknown systematic deviations in some centres. The lack of effect of minocycline is backed up by the lack of effect on secondary measures that are known to relate to negative symptoms, some of which do not involve external raters—eg, the Social Function Scale, which is self-rated, or objective measures such as being in employment. Performance on cognitive function tasks is a known correlate of negative symptoms, but they too were unaffected by minocycline.

The BeneMin study, taken together with the existing literature, suggests that up to 12 months' treatment with minocycline does not improve the symptomatic or functional status of people within 5 years of a diagnosis of schizophrenia. Furthermore, there was little evidence of persisting neurodegeneration or systemic inflammation that the known preclinical actions of minocycline could target. It is possible that such processes occur at an earlier stage, in acute-phase schizophrenia, or in inflammatory or treatment resistant subgroups. However, much firmer biomarker evidence of these pathological processes would seem necessary before further trials of promising anti-inflammatory drugs are done in patients with psychosis. Future studies should also carefully consider the stratification of recruitment and analysis plans, with sufficient numbers of patients included with evidence of an active inflammatory process.

For more on **Catalent** see www.catalent.com

## Data sharing
