# Corrections

**DOI:** 10.1016/S2215-0366(18)30431-0

**Published:** 2018-12

*Deakin B, Suckling J, Barnes TRE, et al. The benefit of minocycline on negative symptoms of schizophrenia in patients with recent-onset psychosis (BeneMin): a randomised, double-blind, placebo-controlled trial.* Lancet Psychiatry *2018; **5**: 885–94*—A footnote has been removed from the author list. This correction has been made to the online version as of Nov 1, 2018.

