# Supplementary material for: The benefit of minocycline on negative symptoms of schizophrenia in patients with recent-onset psychosis (BeneMin): a randomised, double-blind, placebo-controlled trial
Source: Lancet Psychiatry. 2018 Nov;5(11):885–94. doi: 10.1016/S2215-0366(18)30345-6 (PMC6206257; doi:10.1016/S2215-0366(18)30345-6)
Supplement: Supplementary appendix [file mmc1.pdf]

THE LANCET
Psychiatry

Supplementary appendix

This appendix formed part of the original submission and has been peer reviewed. We post it as supplied by the authors.

Supplement to: Deakin B, Suckling J, Barnes TRE, et al. The benefit of minocycline on negative symptoms of schizophrenia in patients with recent-onset psychosis (BeneMin): a randomised, double-blind, placebo-controlled trial. *Lancet Psychiatry* 2018; published online Oct 12. http://dx.doi.org/10.1016/S2215-0366(18)30345-6.

Supplementary Material

Members of the Team not listed as primary authors were:

Amy Bland, Emma Eliasson, Nadim Gire, Catherine George, Farah Lunat, Katrina McMullen, Zoe Rogers Kelly Rushton, Sarndip Sangha, Annya Smyth, Jefter Chuma

Support for the study not listed in the published paper

John Suckling received additional support from the Cambridge NIHR Biomedical Research Centre, the Behavioural and Clinical Neuroscience Institute, University of Cambridge, and the Cambridgeshire and Peterborough NHS Trust Research Strategy Committee.

Bill Deakin contributed additional funding from his NIHR Senior Investigator award for salaries to support the study for Richard Smallman and Amy Bland and to purchase the initial batch of minocycline, an extra treatment cost. In contrast to 10 other NHS trusts, South London and Maudsley NHS Trust did not contribute their share of extra treatment costs.

NHS Trusts involved in the study

Site PIs: Drs Jonathan Hellewell, Tirthankar Mukherjee, Andrew Boardman, Sonia Johnson, Nikola Rahaman, Ilyas Mirza, Remy McConvey.

R&I staff and pharmacists at the following Mental Health NHS and Foundation Trusts: Manchester Mental Health & Social Care NHS Trust; Greater Manchester West MH NHS Foundation Trust; Cheshire and Wirral Partnership NHS Foundation Trust; Lancashire Care NHS Foundation Trust; Pennine Care NHS Foundation Trust; Birmingham and Solihull Mental Health NHS Foundation Trust; Camden And Islington NHS Foundation Trust; North West London Mental Health; West London Mental Health NHS Trust; Barnet, Enfield and Haringey Mental Health NHS Trust South London and Maudsley NHS Foundation Trust; Cambridgeshire and Peterborough NHS Foundation Trust; Lancashire Care NHS Foundation Trust; Royal Edinburgh Hospital, NHS Lothian; NHS Fife, Lynebank Hospital;

OpenCDMS Managers Prof John Ainsworth, Matthew Machin and especially Kathleen Haigh-Hutchinson for their patience in maintaining the database beyond its official close down.

Clinical Research Network staff led by Angie Parker and Moira Winters.

Data Monitoring and Ethics Committee Chair: Prof Emeritus Stephen Cooper (University of Belfast) and members: Dr Carol Gamble (Reader in Medical Statistics, University of Liverpool); Prof Peter Liddle (University of Nottingham); Graham Dunn (University of Manchester, Professor of Biomedical statistics).

Trial Steering Committee Chair: Prof John Geddes (University of Oxford); Prof Robin Jacoby (University of Oxford); Prof Max Birchwood (University of Birmingham); Gerald Wright (service user rep).

Abigail Gee analysed the N-back imaging data as part of her MSc in Neuroimaging for Clinical and Cognitive Neuroscience at the University of Manchester

Table S 1 Schedule of Assessments

Assessment	Who			When						
	CSO	RA	RMO team	Screening	Randomisation	Month 2	Month 6	Month 9	Month 12	Post trial f/u
Case note diagnosis checklist	x	x		x						
Diagnostic and eligibility c'list		x	x	x					x	
DUP			x	x						
MINI interview for psychosis		x		x					x	
Drug treatment history	x	x		x	x	x	x	x	x	x
Body weight & BMI	x	x			x				x	x
BP & HR	x	x		x					x	x
Lab screen	x	x	x	x					x	
Drug screen (urine)	x	x		x			x			
Drug use questionnaire	x	x		x	x	x	x	x	x	x
Pregnancy screen (urine)	x	x		x	x	x	x	x	x	
Inclusion criteria	x	x	x	x						
Exclusion criteria	x	x	x	x						
Withdrawal criteria		x			x	x	x	x	x	
Consent	x	x	x	x						
Consent genetic	x	x	x	x						
Saliva Oragene kit		x			x					
Blood cytokine screen		x	x		x		x		x	x
PANSS		x		x	x	x	x	x	x	x
GAF		x			x	x	x	x	x	x
Social Function Scale	x	x			x		x		x	x
WAIS III (current IQ)	x	x		x					x	x
WTAR (IQ decline)	x	x		x						
Other cognitive tasks	x	x		x					x	x
EPS scales		x			x		x		x	x
Calgary depression scale	x	x			x	x	x	x	x	x
ANNSERS scale (side effects)	x	x			x	x	x	x	x	x
7 point compliance scale		x				x	x	x	x	
MRI screening questionnaire	x	x		x						
MRI scanning		x			x				x	

References for Schedule of Assessments

Sheehan D V, Lecrubier Y, Sheehan KH, *et al.* The Mini-International Neuropsychiatric Interview (M.I.N.I.): the development and validation of a structured diagnostic psychiatric interview for DSM-IV and ICD-10. *J Clin Psychiatry* 1998; **59 Suppl 2**: 22-33;quiz 34-57.

Kay SR, Fiszbein A, Opler L. The positive and negative syndrome scale (PANSS) for schizophrenia. *Schizophr Bull* 1987; **13**: 261–76.

Wechsler D. The Wechsler Test of Adult Reading (WTAR). San Antonio: The Psychological Corporation, 2001.

Addington J, Shah H, Liu L, Addington D. Reliability and validity of the Calgary Depression Scale for Schizophrenia (CDSS) in youth at clinical high risk for psychosis. *Schizophr Res* 2014; **153**: 64–7.

Birchwood M, Smith J, Cochrane R, Wetton S, Copestake S. The Social Functioning Scale. The development and validation of a new scale of social adjustment for use in family intervention programmes with schizophrenic patients. *Br J Psychiatry* 1990; **157**: 853–9.

Blyler CR, Gold JM, Iannone VN, Buchanan RW. Short form of the WAIS-III for use with patients with schizophrenia. *Schizophr Res* 2000; **46**: 209–15.

Joyce EM, Collinson SL, Crichton P. Verbal fluency in schizophrenia: relationship with executive function, semantic memory and clinical alogia. *Psychol Med* 1996; **26**: 39–49.

Schmidt M. Rey Auditory Verbal Learning Test : RAVLT : a handbook. Los Angeles, CA : Western Psychological Services, 1996., 1996.

Table S 2 Means and Standard Deviations for main outcome variables

Range of possible scores in brackets

	**Placebo**			**Minocycline**		
**Measure**	**n**	**mean**	**SD**	**n**	**mean**	**SD**
**Negative Symptoms (7-49)**						
Baseline	104	16.8	5.5	103	17.7	5.9
2m	85	15.1	5.8	83	16.4	5.6
6m	67	15.7	5.8	69	15.8	6.5
9m	62	14.5	4.9	68	15.9	6.3
12m	65	14.2	5.2	62	16.4	6.2
Follow-up	48	14.0	4.9	41	15.6	6.6
**Positive Symptoms (7-49)**						
Baseline	104	17.3	5.3	103	16.3	4.1
2 m	86	14.5	4.8	83	13.8	4.5
6 m	67	14.4	5.2	69	13.4	5.0
9 m	63	13.6	5.0	68	12.8	4.6
12 m	65	14.0	4.8	63	13.4	6.1
Follow-up	48	13.8	5.2	41	13.2	5.3
**Total Symptoms (32-210)**						
Baseline	103	69.3	15.4	103	67.1	13.2
2 m	85	60.1	15.7	83	59.6	14.9
6 m	66	59.4	16.8	69	57.5	15.7
9 m	62	56.8	14.7	68	57.0	14.7
12 m	65	57.1	17.3	62	59.0	17.3
Follow-up	48	55.8	15.4	41	57.7	16.5
**CDSS (0-27)**						
Baseline	103	5.50	4.96	103	5.17	4.27
2m	95	3.40	3.99	94	3.31	3.85
6m	83	3.05	4.17	83	2.60	3.59
9m	80	2.73	3.77	79	3.25	3.78
12m	78	3.12	4.28	76	3.09	3.98
Follow-up	66	2.88	4.43	57	2.49	3.53
**GAF (0-100)**						
Baseline	103	56.2	11.6	102	55.5	9.1
2 m	85	59.5	11.4	83	58.1	11.6
6 m	65	59.6	12.1	68	60.2	13.2
9 m	63	60.8	12.0	67	58.5	12.7
12 m	64	60.4	13.4	60	56.3	14.1

Table S 2 Means and Standard Deviations for main outcome variables (cont'd)

	Placebo			Minocycline		
**Measure**	**n**	**mean**	**SD**	**n**	**mean**	**SD**
**Weight (kg)**						
Baseline	101	86.8	25.3	97	82.6	19.6
12m	58	91.8	28.5	53	88.0	18.2
**BMI**						
Baseline	101	28.7	7.6	96	27.1	6.2
12m	58	30.1	8.5	53	28.7	5.5
**SFS1: Social Engagement / Withdrawal (0-15)**						
Baseline	101	10.2	2.9	103	10.5	3.1
6m	65	11.0	3.2	65	11.0	3.2
12m	63	10.9	3.4	61	10.7	3.7
Follow-up	48	11.5	3.5	41	10.6	3.2
**SFS2: Interpersonal Behaviour/Relations (0-9)**						
Baseline	102	6.6	1.9	103	6.4	1.8
6m	65	7.2	2.0	65	6.8	2.0
12m	63	7.1	2.0	61	6.6	2.2
Follow-up	48	7.1	2.0	41	6.9	2.1
**SFS3: Independence-Performance (0-39)**						
Baseline	102	26.1	6.4	103	26.3	7.5
6m	65	27.4	7.2	65	26.7	8.2
12m	63	27.4	7.0	61	26.3	6.8
Follow-up	48	26.6	6.8	41	26.0	7.4
**SFS4:Recreation (0-45)**						
Baseline	102	17.7	6.01	103	18.2	7.8
6m	65	18.4	7.8	65	17.6	7.3
12m	63	18.4	7.0	61	17.4	7.1
Follow-up	48	17.1	6.8	41	17.4	7.6
**SFS5:Pro-social Activities (0-69)**						
Baseline	102	16.7	10.5	103	16.6	10.3
6m	65	17.3	11.6	65	16.3	9.6
12m	63	17.2	10.8	61	16.5	10.1
Follow-up	48	18.9	11.5	41	15.9	10.0
**SFS6:Independence-Competence (0-39)**						
Baseline	102	34.0	6.2	103	34.8	4.9
6m	65	35.0	4.8	65	34.7	5.4
12m	63	34.8	5.0	61	34.0	5.1
Follow-up	48	35.5	3.9	41	34.0	7.1
**SFS7:Employment/ Occupation (0-10)**						
Baseline	102	4.9	3.0	103	4.7	3.1
6m	65	5.6	3.4	64	4.9	3.3
12m	63	5.9	3.1	61	5.3	3.3
Follow-up	47	5.3	3.3	41	5.4	3,7

Table S 2 Means and Standard Deviations for main outcome variables (cont'd)

	Placebo			Minocycline		
**Measure**	**n**	**mean**	**SD**	**n**	**mean**	**SD**
**Digit Symbol (Raw)**						
Baseline	91	52.8	16.8	95	58.0	16.7
12m	59	58.2	15.8	58	56.1	16.2
Follow-up	47	61.2	15.9	36	62.6	16.2
**Digit Symbol (Scaled)**						
Baseline	91	6.0	2.4	95	6.7	2.7
12m	59	6.8	2.3	58	6.5	2.6
Follow-up	47	7.4	2.4	36	7.4	2.6
**Full-Scale IQ (FSIQ)**						
Baseline	100	89.2	15.9	101	91.2	14.0
12m	61	94.6	16.6	59	93.7	14.2
Follow-up	49	97.0	17.5	38	98.2	16.1
**GMV Left (mm^3)**						
Baseline	88	5669	786	94	5644	723
12m	54	5509	787	45	5593	70
**GMV Right (mm^3)**						
Baseline	88	4581	658	94	4574	583
12m	54	4425	680	45	4543	551
**IL6 (pg/ml)**						
Baseline	100	0.840	0.639	101	0.690	0.458
6m	65	0.902	0.754	57	0.843	0.926
12m	56	0.811	0.623	53	0.793	0.570
**CRP (pg/ml)**						
Baseline	100	3.83	5.45	101	3.08	3.82
6m	65	5.33	9.54	57	4.56	11.23
12m	56	4.40	5.30	51	6.01	18.91

N-Back task performance

Accuracy (percent correct) deteriorated from 0 to 2 back producing a significant main effect of difficulty in a repeated measures ANOVA (table S3 and figure S1). Accuracy improved over 12 months by about 10% at the 2-back level in both groups producing a significant main effect of time. There was no significant interaction between time and treatment

Table S 3 Percent correct responses in N-back task

	Placebo % correct			Minocycline % correct		
**Baseline**	**n**	**Mean**	**SD**	**n**	**Mean**	**SD**
**0 back**	36	77.7	25.4	35	82.3	16.9
**1 back**	36	53.1	27.4	35	55.6	30.1
**2 back**	36	38.7	23.2	35	42.0	26.0
**12 months**						
**0 back**	36	82.3	24.3	35	80.2	26.3
**1 back**	36	59.0	30.4	35	58.9	31.9
**2 back**	36	46.2	26.9	35	49.8	25.9

Figure S 1 Percent correct responses in N-back task

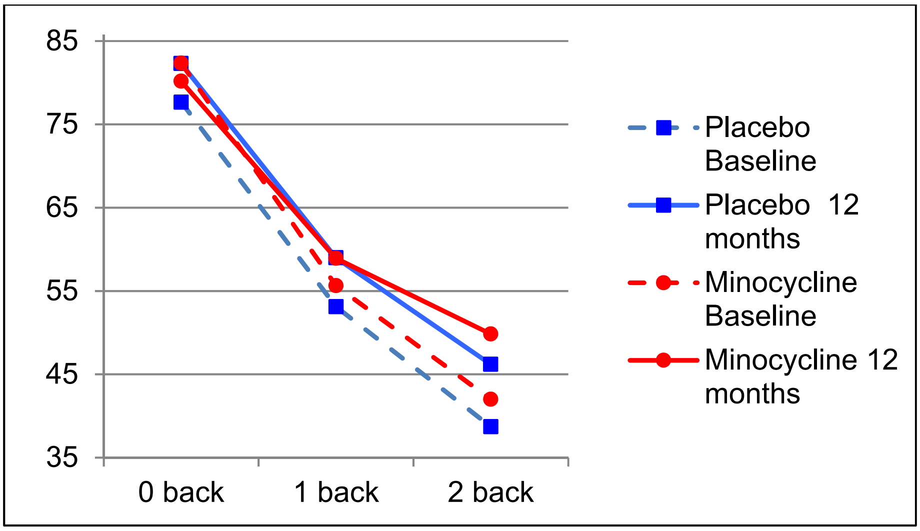

N-Back functional imaging

MRI Pre-processing for VBM and BOLD

i) MPRAGE/SPGR and PD/T2: brain tissue extraction, segmentation via scan-specific tissue priors, non-linear registration to the Montreal Neurological Institute 152 (MNI) standard space, smoothing with an isotropic Gaussian kernel; ii) *EPI:* motion and slice-timing correction, brain tissue extraction, non-linear co-registration with MNI-registered MPRAGE/SPGR, smoothing with an isotropic Gaussian kernel, intensity normalisation, high pass temporal filter. For both MPRAGE/SPGR and PD/T2 images were analysed with sequence-specific voxel-based morphometry (VBM)[1,2]

N-back BOLD MRI Methods

Paired scans (baseline and 12 months) were available for 34 in the placebo group and 36 from the minocycline group. After pre-processing, BOLD response to the 0-,1- and 2-back was estimated at every intracerebral voxel. The following contrasts were then estimated: 1-back and 2-back > 0-back, the overall effect of the working memory task in comparison to the control condition (0-back); and 2-back > 1-back, the differential effect of working memory task difficulty.

Maps of within-group activation for each contrast from all participants at baseline were derived, and demonstrate patterns of activation typical for this well-established task (Figure S2). The regions of interest (ROIs) to test for treatment effects were selected from two meta-analyses of WM in schizophrenia [3,4], focusing on bilateral dorsolateral prefrontal cortex.

Figure S2: Within-group activation patterns at baseline for: (a) 1-back and 2-back > 0-back; (b) 2-back > 1-back.

(a)

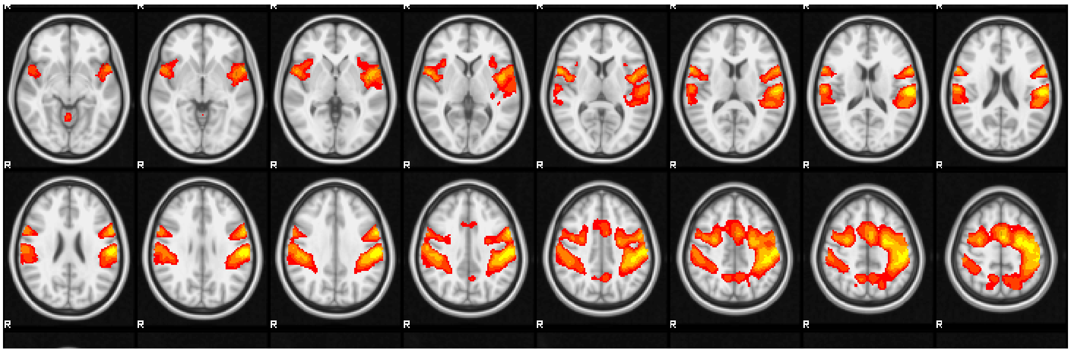

(b)

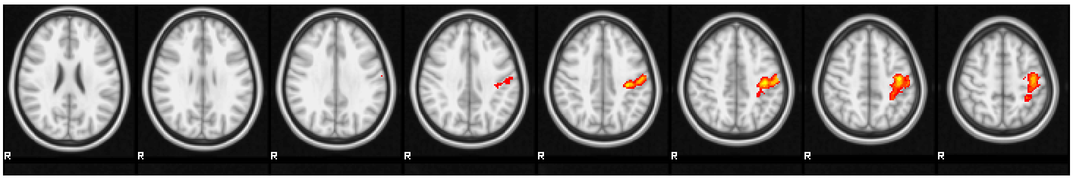

Results

Within-group activation patterns (Figure S2) included the predicted bilateral regions of interest in dorsolateral pre-frontal cortex, anterior cingulate, and inferior parietal lobule.

References

1 Douaud G, Smith S, Jenkinson M, *et al.* Anatomically related grey and white matter abnormalities in adolescent-onset schizophrenia. *Brain* 2007; **130**: 2375–86.

2 Diaz-de-Grenu LZ, Acosta-Cabronero J, Pereira JMS, Pengas G, Williams GB, Nestor PJ. MRI detection of tissue pathology beyond atrophy in Alzheimer’s disease: Introducing T2-VBM. *Neuroimage* 2011; **56**: 1946–53.

3 Glahn DC, Ragland JD, Abramoff A, *et al.* Beyond hypofrontality: A quantitative meta-analysis of functional neuroimaging studies of working memory in schizophrenia. *Hum Brain Mapp* 2005; **25**: 60–9.

4 Minzenberg MJ, Laird AR, Thelen S, Carter CS, Glahn DC. Meta-analysis of 41 Functional Neuroimaging Studies of Executive Function in Schizophrenia. *Arch Gen Psychiatry* 2009; **66**: 811.

7-Point Compliance Scale

The rating was carried out by the RA in a semi-structured interview. The percentage scoring less than 1 or 2 are likely to be non-adherent (see key) and %<3 is the percent of the total number of scores that are 1 or 2. Similarly %> 5 is the percent of the scores that are 6 or 7 which will include adherent participants and some who are over-reporting adherence.

Table S 4 7-Point Compliance Scale

**Allocation**	**Frequency of total adherence scores**								
	**1**	**2**	**3**	**4**	**5**	**6**	**7**	**%<3**	**%>5**
**2 months**									
Placebo	5	2	0	1	4	9	68	8%	87%
Minocycline	8	0	0	1	0	7	72	9%	90%
**6 months**									
Placebo	7	1	1	1	5	8	47	11%	79%
Minocycline	5	1	1	2	3	8	51	8%	83%
**9 months**									
Placebo	8	3	2	2	4	7	42	16%	72%
Minocycline	10	4	0	0	1	6	48	20%	78%
**12 months**									
Placebo	14	3	2	2	3	4	41	25%	65%
Minocycline	11	4	0	0	1	3	46	23%	75%

Key

1 = Complete refusal
2 = Partial refusal or accepts only minimal dose
3 = Accepts only because compulsory, or very reluctant/requires persuasion, or questions need often (e.g. once every two days)
4 = Occasional reluctance (e.g. questions need once a week)
5 = Passive acceptance
6 = Moderate participation, some knowledge and interest in medication and no prompting required
7 = Active participation, readily accepts, and shows some responsibility for regimen

EPSE and Adverse Events

Table S 5 Parkinsonism: mean Simpson Angus EPSE scale total scores (0-40)

	Placebo					Minocycline				
**Measure**	**n**	**mean**	**SD**	**Min**	**Max**	**n**	**mean**	**SD**	**Min**	**Max**
Baseline	102	1.60	2.42	0	15	101	1.60	2.42	0	13
6m	66	1.29	2.01	0	10	65	1.60	2.07	0	7
12m	61	1.25	2.59	0	13	61	1.72	2.46	0	10
follow-up	47	1.04	2.27	0	13	41	1.83	2.52	0	9

Table S 6 Antipsychotic Non-Neurological Side-Effects Rating Scale (ANNSERS)

	Placebo			Minocycline		
**Measure**	**n**	**mean**	**SD**	**n**	**mean**	**SD**
Baseline	103	13.36	7.09	102	11.49	7.30
2 months	80	7.62	6.96	80	6.39	5.97
6 months	59	6.75	6.92	67	6.57	5.90
9 months	58	6.10	6.19	62	6.70	6.06
12 months	60	6.90	6.62	58	7.29	6.77
follow up	44	5.51	5.96	38	6.70	7.34

Table S 7 Akathisia: distribution of BARS global scores

	Placebo					Minocycline				
**Score**	**0**	**1**	**2**	**3**	**n**	**0**	**1**	**2**	**3**	**n**
Baseline	83	7	12	1	103	77	13	9	1	100
12 mth	51	3	9	0	63	49	6	4	1	60

Table S 8 Tardive dyskinesia: distribution of AIMS scores

	Placebo							Minocycline						
**Score**	**0**	**1**	**2**	**3**	**4**	**5**	**n**	**0**	**1**	**2**	**3**	**4**	**5**	**n**
Baseline	89	9	1	2	0	0	103	86	8	1	1	0	3	100
12 mth	58	2	3	0	0	0	63	54	2	2	0	0	1	60

Table S 9 Adverse events and reactions, and serious adverse events

Event Type	Placebo	Minocycline
**Adverse Events (AEs)**		
Total number of AEs	67	60
Psychiatric events (MedDRA cat 19)	16	8
**Adverse Reactions (ARs)**		
Rash	3	2
GI upset	6	2
Headache	2	1
Total ARs	11	5
**Severe Adverse Events (SAEs)**		
Total number of SAEs	11	18
Psychiatric hospitalisation events (patients)	10 (6)	15 (10)
Admission for abdominal pain (patients)	0 (0)	3 (2)
DVT	1	0

NB Effect of treatment on PANSS negative and positive in univariate ANOVAs for each visit controlling for baseline did not reveal significant effects when relapsers were excluded.
